# A rapid method for isolation of genomic DNA from food-borne fungal pathogens

**DOI:** 10.1007/s13205-016-0436-4

**Published:** 2016-06-06

**Authors:** S. Umesha, H. M. Manukumar, Sri Raghava

**Affiliations:** Department of Studies in Biotechnology, University of Mysore, Manasagangotri, Mysore, 570006 Karnataka India

**Keywords:** CTAB, TPCI, MW, S-CCI, PCR, DNA

## Abstract

Food contaminated with fungal pathogens can cause extremely harmful effects to human even when present in low concentrations. Researchers now pay more attention towards rapid DNA extraction for the quick screening, which is highly demanded in diverse research field. Molecular description of many fungal species is identified by different molecular characteristics. Hence, the efficient isolation of genomic DNA and amplification using PCR is a prerequisite for molecular characterization. Here, we used an improved Sodium dodecyl sulfate-Cetyltrimethyl ammonium bromide-Chloroform-isoamyl alcohol method by combining Sodium dodecyl sulfate with cetyl methylammonium bromide without addition of proteinase K, RNase K, and β-mercaptoethanol. To analyze the quality of recovered DNA, this method was compared with the other four routine methods. The present method has been chosen in the study as a preferred method because of easy adaptation to routine laboratories/food industries considering its rapid, sensitivit,y and cost effectiveness involved in the method.

## Introduction

In under-developed countries, one of the leading causes of illness and death is due to food-borne pathogens, which accounts approximately up to 1.8 million people annually (Bisha and Brehm-Stecher 2010). To ensure food safety, rapid detection of pathogenic organisms causing food-borne illness is a basic requirement. Plating methods have been replaced by more rapid and sensitive methods, such as Fluorescence *In*-*Situ* Hybridization (FISH) (Chattopadhyay et al. 2013), Enzyme Linked Immuno-Sorbent Assays (ELISA) (Naravaneni and Jamil 2005), Polymerase Chain Reaction (PCR) (Jaykus 2003), and Real-Time PCR (RT-PCR) (Wolffs et al. 2004). However, the prerequisite for all these methods is a high-quality DNA from the pathogen. Various procedures are being used in these contests, but these protocols are mainly suited for specific groups with known morphologies and not for versatile fungal groups. Therefore, DNA extraction is a very critical step, as it eliminates unwanted interfere substances and ensures consistency in the nucleic acid test results (Bolano et al. 2001).

It is a well-known fact that extraction of pure DNA from fungi is very difficult. Reports exist that DNA extracted from *Neotyphodium lolii* (Christensen et al. 1993) using methods of Raeder and Broda (1985) and Byrd et al. (1990) that was neither digested nor amplified by restriction enzymes and *Taq* polymerase, respectively. This was mainly due to cross contamination of fungal polysaccharides or agar inoculum taken from the plates. Recently commercial kits have been popularized (Dieguez et al. 2009), because DNA can be extracted easily within a day. Since the available kit-based and other DNA extraction methods are time consuming, and expensive. researchers have also worked with different approaches to explore the best manual method to extract fungal DNA, such as CTAB (Petrisko et al. 2010) with organic solvents, lyticase, phenol–chloroform, and isoamylic alcohol (Shin-ichi and Takuma 2010) chelex (Hennequin et al. 1999) and the urea chelex method (Mseddi et al. 2011) for some fungal mycelia and most fungal spore samples remain undesirable.

In this present paper, Sodium dodecyl sulfate-Cetyltrimethyl ammonium bromide-Chloroform-isoamyl alcohol (S-CCI) method was used for the isolation of food-borne fungal genomic DNA. Sodium dodecyl sulfate (SDS) is a strong anionic detergent, which disrupts non-covalent bonds in the proteins and denature or losing its confirmation. Cell membrane composed of proteins and lipids in varying percentage. When external force/chemicals applied, then membrane act against defence and become destabilized this leads to breakdown of nuclear envelop and expose of nuclear material to the outer environment. In addition, removing the membrane barriers helps to release the DNA from histones and other DNA-binding proteins by denaturing them. The CTAB extraction method originally developed by Doyle and Doyle in 1987, and later, it was modified to remove polysaccharide, polyphenols, and other secondary metabolites. The superfluous quantities of cellular proteins were eliminated by triple extended treatment with chloroform-isoamyl alcohol. In addition to the removal of proteins, this treatment also helps to remove different coloring substances. Importantly, CTAB is probably the only compound that can separate partial nucleic acids from polyphenols. The polyphenolic compounds may severely inhibit downstream DNA/RNA reactions. Chloroform-isoamyl alcohol is a type of liquid detergent disrupts the bonds that hold the cell membranes by dissolving proteins, lipids, and then form complexes to precipitate out of the solution.

The modified extraction protocol was designed based on four factors, to maximize the DNA yield, minimize the time, and avoid the use of expensive chemicals in extraction steps, and DNA should be amenable to several downstream enzymatic applications, such as PCR amplification. Therefore, the objective of this study was to compare existing extraction methods to our modified S-CCI protocol for high-quality total DNA from fungi, such as *Aspergillus niger, Aspergillus flavus, Aspergillus fumigates, Acremonium strictum, Bipolaris cyanodontis,* *Colletotrichum gloeosporioides, Fusarium equiseti, Fusarium oxysporum, Penicillium*, and *Trichoderma*.

## Materials and methods

### Sample collection and Isolation of mycoflora

Different vegetarian and non-vegetarian food samples were collected from different locations of Mysore region and sterilized with 3 % sodium hypochlorite, 70 % ethanol, and followed by repeated washings with sterile distilled water. All samples were subjected to non-selective medium, potato dextrose agar (PDA), and incubated at 25 ± 2 °C for 7 days. Then, fungal cultures were identified based on morphological characteristics using standard book by Mathur and Kongsdal, (2003) and pathogens were purified by culturing onto new plates and individual cultures were inoculated into potato dextrose broth (PDB) followed by incubation for 10 days at 25 ± 2 °C. Grown mycelial mat was freeze dried (at −20 °C), lyophilized (at −50 °C), and further DNA isolation and purification procedure were carried out for these samples.

### Reference strain

The reference *Aspergillus brasiliensis* (MTCC-1344) fungal strain was kindly gifted by Ananda, A. P., Department of Microbiology, Ganesh consultancy, and analytical services, Mysore. Cultures acquired from Microbial Typing Culture Collection, Chandigarh, India, used as positive control for experimental study, and culture was revived and grown as per protocol prescribed.

### Genomic DNA extraction methods

#### CTAB-phenol–chloroform-isoamyl alcohol method

200 mg of lyophilized mycelial mat were grounded with pestle and mortar using 500 µL of [*CTAB*-phenol–chloroform-isoamyl alcohol method (CTABPCI)] extraction buffer (200-mM Tris–HCl (pH 8.0), 25-mM EDTA (pH 8.0), 250-M NaCl, 10 % CTAB) according to Li and Yao (2005). Transferred to fresh tube and 3-µL proteinase K, 3-µL RNase were added then vortex and incubated for 1 h at 37 °C. After incubation tubes were kept in a water bath for 10 min at 65 °C. After one volume of phenol: chloroform: isoamyl alcohol (25:24:1) was added, solution was thoroughly mixed for 5 min then centrifuged at 12,000 rpm for 5 min. The aqueous clear phase was recovered and mixed with one volume of chloroform: isoamyl alcohol (24:1), centrifuged at 12,000 rpm for 5 min, and the aqueous phase was recovered. Added one volume of ice-cold isopropanol and stored overnight for precipitation of DNA at −20 °C. DNA was recovered by centrifugation at 10,000 rpm for 5 min and DNA was precipitated with absolute ethanol. The DNA was then rinsed twice with 1 ml of 70 % ethanol and resuspended in 200µL of deionized water or 1X TE [200-mM Tris–HCl (pH 8.0), 20-mM EDTA (pH 8.0)] buffer for further use.

#### Tris-phenol–chloroform-isoamyl alcohol method

Lyophilized 200 mg of fungal cultures were transferred to 2-ml fresh tubes. 500 µL of TPCI (*Tris*-*phenol*–*chloroform*-*isoamyl alcohol*) extraction buffer (10 mM Tris–HCl, pH 8.0, 100 mM NaCl, 25 mM EDTA, 0.5 % SDS), and 3 µL proteinase K was added, vortexed, and incubated for 1 h at 37 °C. Tubes were kept in a water bath for 10 min at 65 °C and centrifuged at 10,000 rpm for 10 min. Upper liquid part was carefully transferred to a new tube and one volume of phenol: chloroform: isoamyl alcohol (25:24:1) mixed thoroughly for 5 min and centrifuged at 10,000 rpm for 5 min. The aqueous phase was recovered, added 3-µL RNase, and incubated for 30 min. Mixed with one volume of chloroform: isoamyl alcohol (24:1), and tubes were centrifuged at 10,000 rpm for 5 min. Again, the aqueous phase was recovered and one volume of ice-cold isopropanol was added and the tubes were stored overnight at −20 °C. After centrifugation at 10,000 rpm for 5 min, the pellet was recovered and DNA was precipitated with absolute ethanol. The DNA was then washed twice with 1-ml 70 % ethanol and resuspended in 200 µL of deionized water as per Silva et al. (2014).

#### Microwave method

The cultures were collected and grown as described above, and the gDNA was purified by the microwave (MW) method as reported previously by Bollet et al. (1995). 200 mg of fungal mat was rinsed with 1-ml TE buffer, centrifuged, and lysed with 100-µL TE buffer and 50-µL 10 % SDS. Incubated for 30 min at 65 °C and centrifuged to remove the supernatant. The cell pellet was placed in a microwave oven, heated two times for 1 min at 900 W. The pellet was then resuspended in 200-µL TE buffer with one volume of phenol: chloroform: isoamyl alcohol (25:24:1) for 15 min. The aqueous phase was recovered by centrifugation; the DNA was precipitated with 95 % ethanol and centrifuged at 12,000 rpm for 20 min. Then, the DNA was rinsed with 1-ml 70 % ethanol and resuspended in 200-µL deionized water as previously described.

#### Inexpensive method

Fungal cultures were collected and grown as described above for inexpensive DNA extraction (IM) method as reported previously by Prabha et al. (2012). 200 mg of lyophilized mycelia mat were rinsed with 1-ml TE buffer, vortexed for 10 s, tubes were kept at room temperature for 30 min. After centrifugation (10,000 rpm for 10 min), supernatant was transferred to a new tube, then, the equal volume of phenol: chloroform (24:1) was added, mixed properly, and centrifuged at 13,000 rpm for 2 min. Finally, supernatant was collected into separate tube, 300 µL of ice-cold isopropanol was added and gently mixed in tubes inversely. The reaction mixture was incubated at −20 °C for 30 min. DNA was recovered by centrifugation at 13,000 rpm for 5 min and pellet was washed with ice-cold 70 % ethanol and air dried for 15 min at room temperature. Finally, pellet was resuspended in 100 µL of sterile water and stored at −20 °C for further use.

#### SDS-CTAB-chloroform-isoamyl alcohol (modified method)

200 mg of lyophilized mycelial powder was taken and transferred to 2-ml eppendorf tube. 500 µL of SDS-CTAB-chloroform-isoamyl alcohol (S-CCI) extraction buffer (250-mM Tris–HCl (pH 8.0), 20-mM EDTA (pH 8.0), 200-M NaCl, 10 % CTAB, 0.15 % SDS) was added and vortexes and boiled for 10 min at 50 °C and centrifuged at 10,000 rpm for 10 min. Carefully, upper liquid part was pipetted out and one volume of chloroform: isoamyl alcohol (23:2) was added then mixed for 1 min and centrifuged at 10,000 rpm for 5 min. The aqueous phase was recovered and mixed with one volume of ice-cold isopropanol, and tube was turned upside down for 1 min to precipitate DNA. Tubes were centrifuged at 10,000 rpm for 2 min to recover the pellet and washed with 500 µL of absolute ethanol, then centrifuged at 10,000 rpm for 1 min. DNA was air dried and resuspended in 200-µL deionized or TE buffer for further use.

### DNA quantification and quality determination

Extracted genomic DNA concentration and purity were determined by NanoDrop spectrophotometer (Thermo Fisher Scientific, USA). Concentration was recorded in ng/µL, and purity of DNA is based on the ratio of the optical density (OD) at the wavelength of 260 nm and 280 nm. The quality of the DNA yielded by each method was determined by gel electrophoresis in a 0.7 % agarose gel.

### Restriction digestion

According to Devi et al. (2015) with slight modifications, quality of the genomic DNA was validated using *Eco* RI (Fermentas, Germany) restriction enzyme. The S-CCI method extracted DNA was subjected for enzyme digestion (one sample per triplicate) to check the suitability of the DNA for downstream applications. Reaction volume set for 20 μL contain 5 μL of 10X assay buffer [1X buffer composition: 10-mM Tris–HCl pH 8.0; 5-mM MgCl_2_;100-mM KCl; 0.02 % TritonX-100; 0.1-mg/mL BSA], 1–2 μg of the DNA template with 20 U of the enzyme, and reaction volume make up using 1X enzyme buffer. Then, reaction carried out at 37 °C for 20–60 min in water bath. Finally, reaction was stop by heat inactivating the enzyme at 65 °C for 10 min and 5 μL of the digested products were analyzed on 0.8 % agarose gel along with 1 Kb DNA ladder.

### Polymerase Chain Reaction

Polymerase Chain Reaction (PCR) assay was performed using ITS rDNA primers. According to Gonzalez et al. (2008), PCR amplification reaction was performed using ITS1 F (TCCGTAGGTGAACCTGCGG) and ITS4 R (TCCTCCGCTTATTGATATGC) primer set. Amplification was carried out in 0.2-ml tube and reaction mixture containing 2.5 µL of 80–100 ng of genomic DNA, 1 µL of 20 pmol of each primer, and 20 µL of Dream Taq Green PCR master mix (Containing: 0.25 mM each dNTP, 2 mM MgCl_2_ and Taq DNA polymerase) purchased from (Thermo scientific, India). The PCR was performed in a master gradient thermal cycler (LABNET, NJ, USA) using the following conditions: initial denaturation at 94 °C for 5 min; 30 cycles of denaturation for 1 min at 94 °C, annealing for 1 min at 52 °C, initial extension for 1 min at 72 °C, and final extension of 10 min at 72 °C, followed by cooling at 4 °C until the samples were recovered. The amplified PCR amplicons was confirmed through gel electrophoresis using 1 % agarose gel.

## Results and discussion

**Fig. 1:**
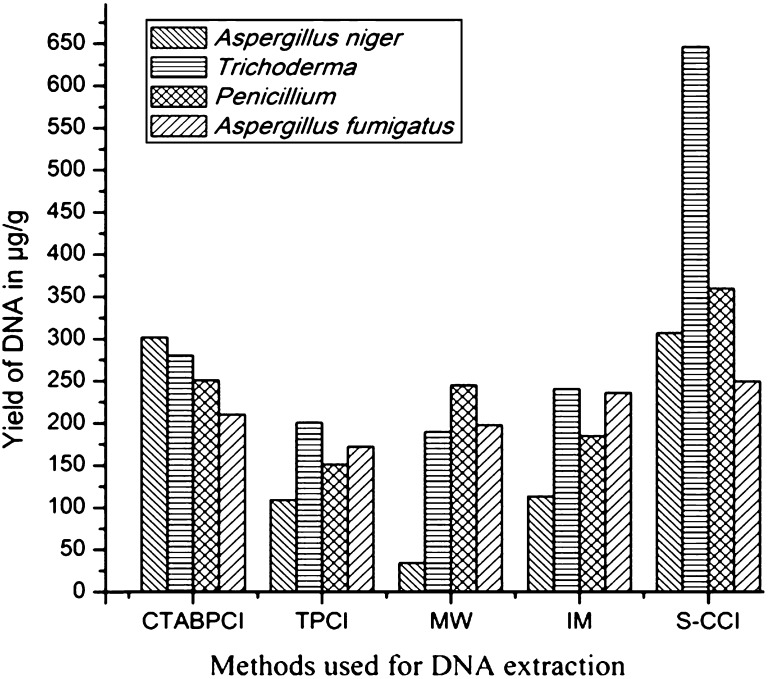
Yield of DNA in different DNA extraction methods. Different fungal genomic DNA was prepared by CTABPCI, TPCI, MW, IM, and modified S—CCI methods. Highest DNA yield was obtained by the S-CCI method from *Aspergillus niger, Trichoderma, Penicillium*, and *Aspergillus fumigates* compared to the other methods of DNA extraction. Among different DNA extraction methods, the S-CCI method showed maximum yield of DNA

Due to the presence of cell wall in fungi, it interferes and hinders the efficiency of DNA extraction from the conventional extraction methods (Maaroufi et al. 2004). After several repetitions, we have optimized rapid and inexpensive method to isolate of fungal DNA by slight modifications in the existing CTAB buffer constitution and steps involved in DNA extraction (Rogers 1989). The yield and purity of genomic DNA obtained from all four extraction methods are depicted in Table 1. Different species of microorganisms having its own varied membrane structural organization with unique sets of protein to carry out the specialized functions (Arachea et al. 2012). In the present study, four different detergents-based protocol for disruption of membrane structure and removal of proteins (irreversibly from the cell) have been compared. The high DNA yield was obtained in S-CCI protocol (645.45 μg/g sample for *Trichoderma*) followed by CTABPCI, TPCI, MW, and IM in descending order was represented in Table 1. It is evident from the data that protocol IM recovered very less yield compared to the other methods. In Fig. 1, the highest yield of DNA extracted by different methods was depicted.

**Table 1:** Genomic DNA yield from different fungal pathogens using different extraction methods

Sl. no	Method	Pathogens	Yield (μg DNA/g sample)	Purity (A_260_/A_280_)
1	CTABPCI	*Aspergillus niger*	301.25	1.90
		*Aspergillus flavus*	246.95	1.67
		*Bipolaris cyanodontis*	210.52	1.92
		*Fusarium oxysporum*	239.57	2.01
		*Penicillium*	250.45	2.10
		*Trichoderma*	280.00	1.96
		*Fusarium equiseti*	219.95	1.87
		*Acremonium strictum*	195.30	2.00
		*Colletotrichum gloeosporioides*	236.20	1.98
		*Aspergillus fumigatus*	210.30	1.65
2	TPCI	*Aspergillus niger*	108.85	1.75
		*Aspergillus flavus*	33.97	1.94
		*Bipolaris cyanodontis*	158.62	2.01
		*Fusarium oxysporum*	97.27	1.84
		*Penicillium*	151.00	1.99
		*Trichoderma*	200.45	2.10
		*Fusarium equiseti*	51.97	2.21
		*Acremonium strictum*	167.52	2.10
		*Colletotrichum gloeosporioides*	172.13	1.69
		*Aspergillus fumigatus*	171.85	1.85
3	MW	*Aspergillus niger*	34.67	1.40
		*Aspergillus flavus*	189.05	2.10
		*Bipolaris cyanodontis*	110.25	1.78
		*Fusarium oxysporum*	70.32	1.75
		*Penicillium*	244.50	1.98
		*Trichoderma*	189.67	2.10
		*Fusarium equiseti*	185.67	1.89
		*Acremonium strictum*	46.75	1.78
		*Colletotrichum gloeosporioides*	105.52	1.70
		*Aspergillus fumigatus*	197.39	1.50
4	IM	*Aspergillus niger*	113.21	1.84
		*Aspergillus flavus*	211.32	1.98
		*Bipolaris cyanodontis*	78.05	2.20
		*Fusarium oxysporum*	127.60	2.01
		*Penicillium*	184.72	1.45
		*Trichoderma*	220.42	1.79
		*Fusarium equiseti*	152.60	1.99
		*Acremonium strictum*	67.75	2.00
		*Colletotrichum gloeosporioides*	211.00	1.89
		*Aspergillus fumigatus*	235.47	1.98
5	S-CCI	*Aspergillus niger*	306.47	1.99
		*Aspergillus flavus*	596.15	1.93
		*Bipolaris cyanodontis*	314.80	2.01
		*Fusarium oxysporum*	390.02	2.00
		*Penicillium*	359.47	1.70
		*Trichoderma*	645.45	1.83
		*Fusarium equiseti*	354.92	2.01
		*Acremonium strictum*	253.53	1.72
		*Colletotrichum gloeosporioides*	305.07	1.69
		*Aspergillus fumigatus*	249.36	1.78

Different fungal cultures were used for extraction of DNA by CTABPCI, TPCI, MW, and IM compared to modified S-CCI method in this study. Yield and purity of established S-CCI method were compared to other methods. Each values of yield and purity mentioned are average of triplicate assay carried out for all the fungal pathogens used

**Fig. 2:**
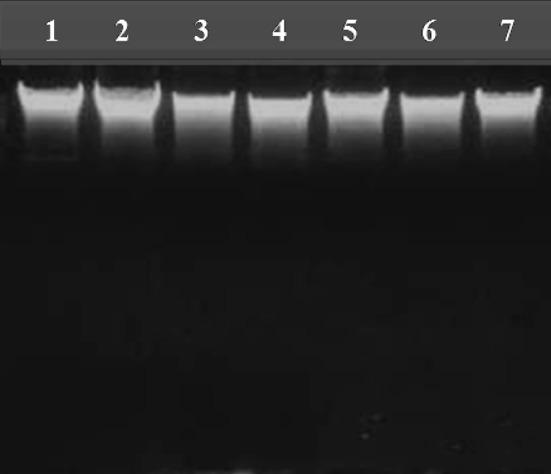
Genomic DNA profile of different fungi, extracted using S-CCI method on 0.7 % agarose gel. Genomic DNA was extracted from different fungi using S-CCI method and electrophoresed on 0.7 % agarose gel. S-CCI method showed clear DNA profile of all fungal organism studied. *Lane 1*. *Aspergillus niger, 2. Aspergillus flavus, 3. Bipolaris cyanodontis, 4. Fusarium oxysporum, 5. Penicillium, 6. Trichoderma*, and *7. Fusarium equiseti*

**Fig. 3:**
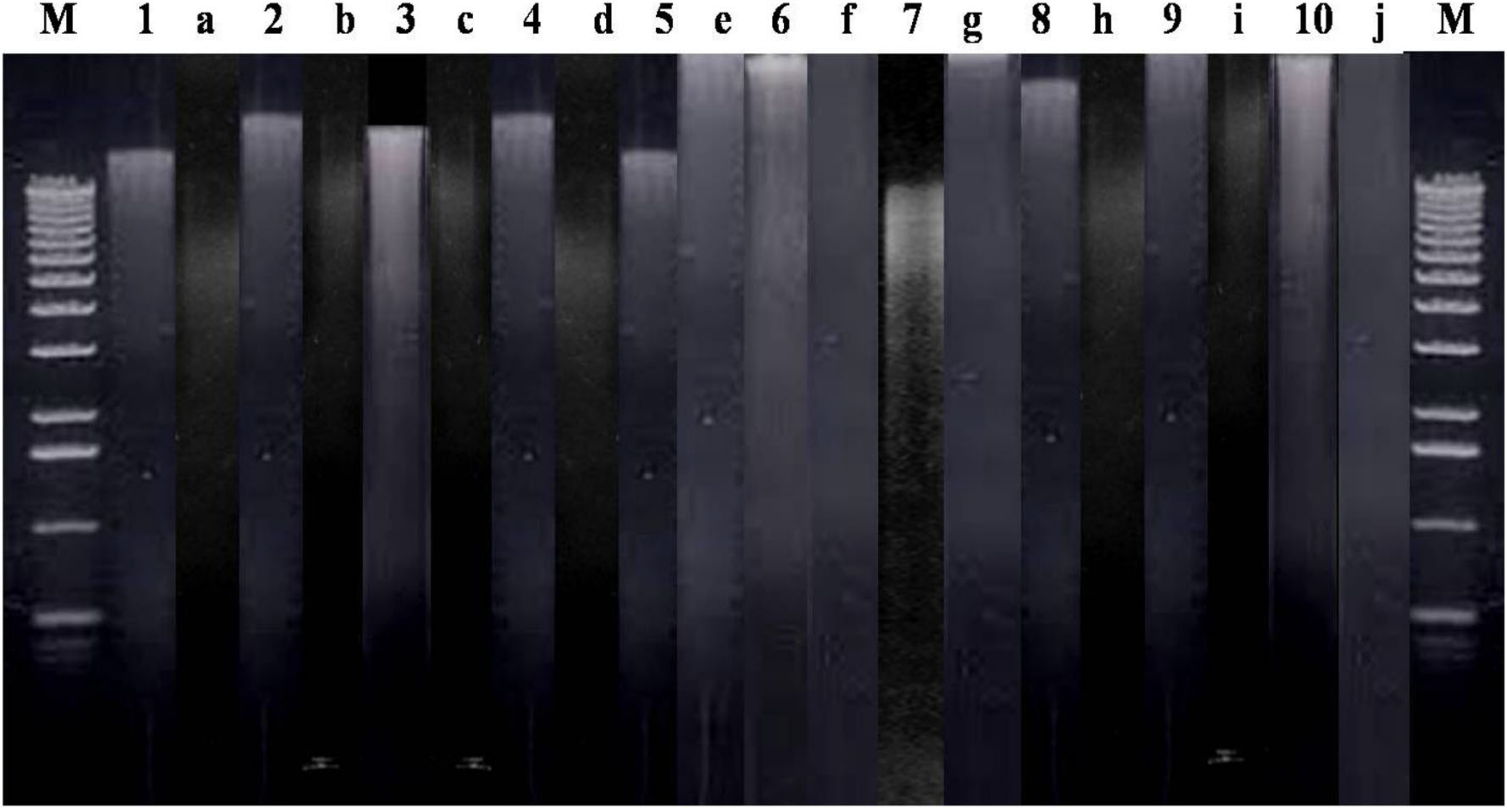
Gel electrophoresis of partial restriction digestion of the genomic DNA extracted by the S-CCI method using restriction enzyme *Eco* RI. Compared to CTABPCI, TPCI, MW, and IM DNA extraction methods, S-CCI exhibited its downstream application showing less contamination, while extracting DNA of fungal pathogens. *Lane 1*. *Aspergillus niger, 2. Aspergillus flavus, 3. Bipolaris cyanodontis 4. Fusarium oxysporum, 5. Penicillium, 6. Trichoderma 7. Fusarium equiseti*, *8. Acremonium strictum, 9. Colletotrichum gloeosporioides*, and *10. Aspergillus fumigatus* followed by restriction digestion products (*a*–*j*), respectively, on 1 % agarose gel. *Lane M* 1 kb DNA ladder

The purity of DNA from fungal pathogens using S-CCI protocol was followed according to the Ki et al. (2007); Desloire et al. (2006) and results depicted in Table 1. The S-CCI extracted genomic DNA of fungal pathogens was run on 0.7 % agarose gel (Fig. 2), to compare the quality of DNA. The CTAB method has been primarily developed for extraction of DNA from plant tissues. This superior method helps in removing unwanted carbohydrates associated during plant DNA extraction (Goltapeh et al. 2007; Petrisko et al. 2008a, b). Muller et al. (1998) reported high-speed cell disruption extraction produced a significantly greater yield from fungi. Fredricks et al. (2005) used a FDNA followed the same principle accordance with the Muller, and it is comparable to our S-CCI protocol. According to Liu et al. (2011), FPFD (Fast Purification of Fungal DNA) method also promisingly explained satisfactory recovery of fungal DNA from FPFD experimental performance. FPFD method does not require organic extraction, and this method meet the needs of the routine screening of fungal pathogens. All methods except TPCI and MW method highlighted cross contamination of protein in the sample were depicted in Table 1. Values of an OD ratio factor of the purity of DNA; below 1.8 indicates protein contamination while above 1.8 indicates contamination of RNA (Samuel et al. 2003). The purity of DNA was compared among different methods that were represented in Table 1. The purity of the all the DNA was further confirmed by digesting the genomic DNA using restriction enzyme *Eco* RI. Then, photograph was showing the banding pattern of digested DNA along with genomic DNA in Fig. 3, it is comparable to the Ajay et al. (2008).

Important features of this S-CCI protocol are: 1. The method works well with all species of fungus to extract genomic DNA. 2. Yields high quality of DNA from mycelium without fragmentation of DNA. 3. Very simple, cost effective, and requires less manpower. 4. Compared to kit-based methods, this method is quite fast with less extraction steps and chemicals required. According to Khan and Yadav (2004), excessive or above 0.01 % of SDS residues in the sample cause denaturation of the Taq DNA polymerase or crass act or inhibit the PCR amplification; so, use of less percentage of SDS is must required. Therefore, in our study, we have used 0.15 % of SDS in the S-CCI method; hence, no cross acting on PCR was observed. So, we recommend not more than 0.15 % of SDS (higher may affect the PCR) to prepare the S-CCI extraction buffer.

**Fig. 4:**
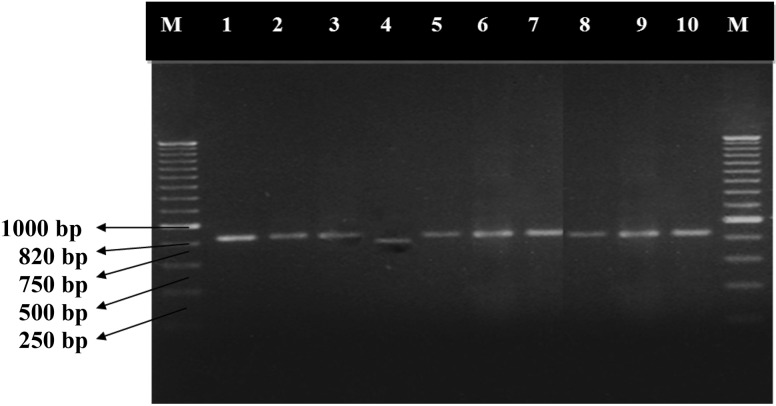
Amplified PCR profile of different fungal ribosomal DNA extracted from the S-CCI method. Among the different (CTABPCI, TPCI, MW, and IM) methods, downstream application of PCR was well-defined by the S-CCI method. The PCR was performed using ITS1 and ITS4 primers and electrophoresed on 1 % agarose gel. *Lane 1*. *Aspergillus niger, 2. Aspergillus flavus, 3. Bipolaris cyanodontis, 4. Fusarium oxysporum, 5. Penicillium, 6. Trichoderma, 7. Fusarium equiseti*, *8. Acremonium strictum, 9. Colletotrichum gloeosporioides,* and *10. Aspergillus fumigatus*

**Fig. 5:**
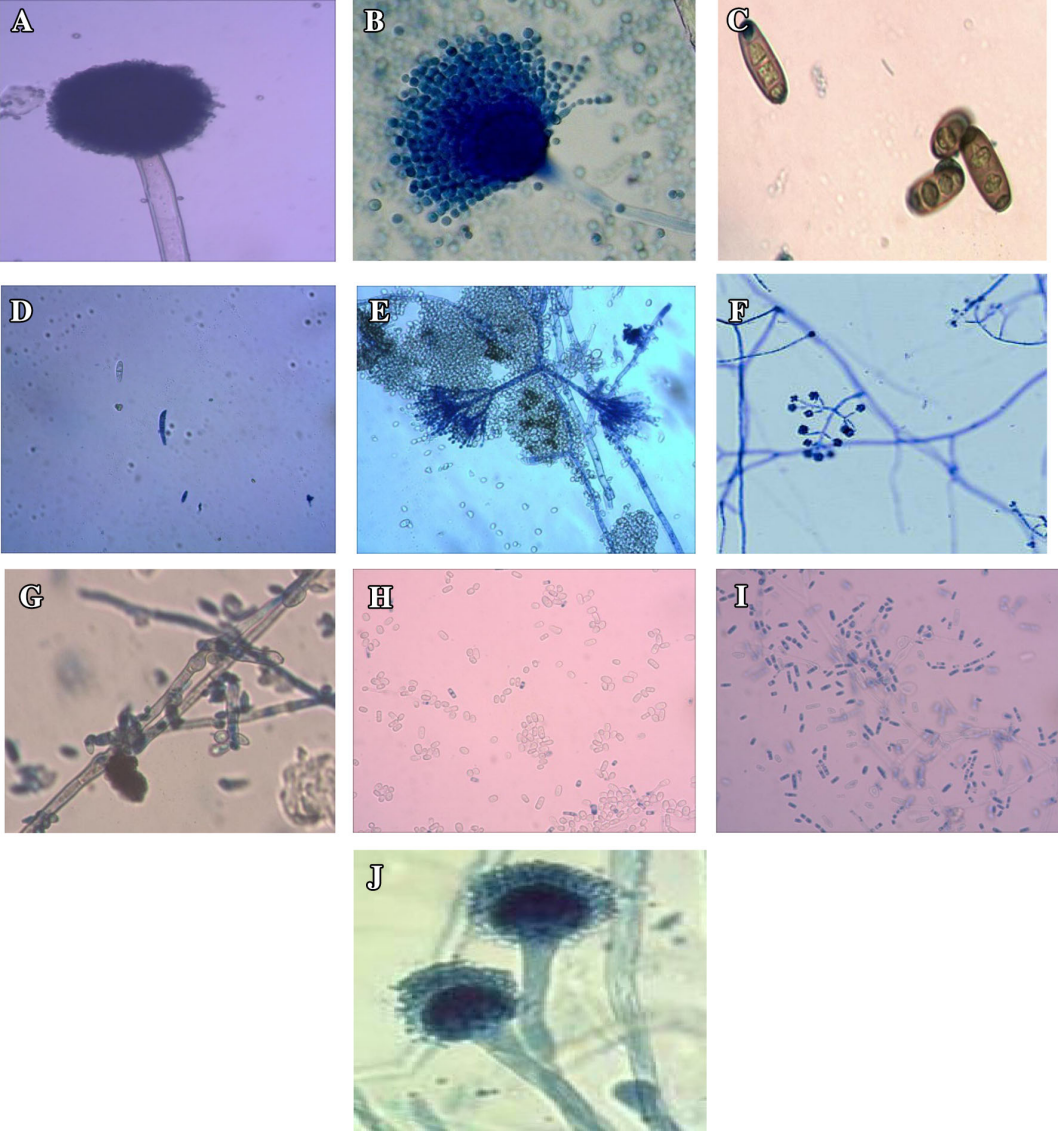
Morphological characterization of fungal pathogens under compound microscope. Different fungal pathogens cultured on PDA medium were subjected to morphological characterization under compound microscope. **a** *Aspergillus niger,* **b** *Aspergillus flavus,* **c** *Bipolaris cyanodontis,* **d** *Fusarium oxysporum,* **e** *Penicillium,* **f** *Trichoderma,* **g** *Fusarium equiseti,* **h** *Acremonium strictum,* **i** *Colletotrichum gloeosporioides*, and **j** *Aspergillus fumigatus*

To check the quality of fungal genomic DNA, fungal Internal Transcribed Spacer (ITS) specific universal primers ITS1F and ITS4R were used for amplification of the fungal rDNA region. Figure 4, represents ITS primer amplified PCR products of S-CCI extracted DNA of different fungi (morphological characteristics were depicted in Fig. 5), which have been well resolved in 1 % agarose gel and compared to 100-bp ladder. Confirmed that the DNA sample are free of polyphenols and polysaccharides, which are inhibitory agents hidden in the sample known to inhibit restriction endonucleases (RE) and Taq DNA polymerase according to Moyo et al. (2008). Restriction enzyme digested samples in Fig. 3 also confirmed that isolated DNA was amenable for downstream applications was successfully explained in the paper is comparable to the Ajay et al. (2008).

Fredricks et al. (2005) compared two important human fungal pathogens (*Aspergillus fumigatus* and *Candida albicans*) using six kit-based DNA extraction protocol. Among them, MPY (Master Pure Yeast DNA purification kit) and FDNA protocol produced a good result yielding the high amount of fungal DNA. In comparison to kit-based protocol and available existing DNA extraction methods, S-CCI executionproved that it is more advantageous, yielding more fungal DNA over kit-based MPY, FDNA, and compared four methods in this study. Fredricks method of DNA isolation has a major drawback of low yield of DNA. To overcome this drawback, necessay link was highlighted in the S-CCI method by altering chemical compositions and steps important for the recovery of fungal DNA method was optimized in this paper.

Some of the species grow as unicellular that reproduce by budding or binary fission in case of yeasts. However, dimorphic fungi can grow in between a yeast phase and a hyphal phase in response to environmental conditions. Plants cell wall composed of glucans and exoskeleton of arthropods made by chitin. But in the case of fungal cell, celwall is composed of both chitin and glucans. The only organisms combine both these structural molecules in their cell wall. Except plants and oomycetes, cell walls of fungi do not contain cellulose. Fungi are one of the most successful species and are distributed worldwide. The cell wall of a fungus is an intriguing component. It determines the shape of the cell and also protects cell from harsh environment.

The fungal kingdom is very diverse in nature, species growing as unicellular, or hyphae with branches helps in production of remarkable assortment of spores and other reproductive structures. For every stage of fungal development, the shape and close association between protein and other polymers present integrity that present in the cell wall of the fungus is play a very important role in the dependent by giving mechanical strength, intern which performs a wide range of fundamental roles during the communication of the fungus with its environment (Gooday 1995).

We isolated different (*Aspergillus niger, Aspergillus flavus, Bipolaris cyanodontis, Fusarium oxysporum, Penicillium, Trichoderma, Fusarium equiseti, Acremonium strictum, Colletotrichum gloeosporioides,* and *Aspergillus fumigates*) fungal pathogens from food sources and subjected to DNA extraction using different protocols by comparing our fast DNA extraction method to show the efficiency and for downstream applications of DNA. Critically, the fungal wall is a multifarious organization by tranquil typically of chitin, 1,3-β- and 1,6-β-glucan, mannan, and proteins.

Primarily, monomers of β-1,4-linked N-acetylglucosamine and β-1,3-linked glucose repeats associated with formation of chitin and glucan polymers, respectively. However, considering this point of view and look up the structural association in fungal cell wall, there is abundant quantities of branched 1,3- β -,1,6- β –glucan, and also evidence of Klis et al. (2002), presence of extensive cross-linking between chitin, glucan, and other wall components. Furthermore, the highly dynamic structure of cell wall involved in constant revolutionize during cell division, spore germination, branching of hyphal structures, and septum pattern in filamentous fungi. Maybe, these are activities that depend on number of hydrolytic enzymes intimately allied with the cell wall of the different fungal species. Fungal genome contains multiple glucanase/glucanosyltransferase-encoding genes. One of the possible results of gene disruption studies states that several of these enzymes have roles during development of cell-wall architecture in yeasts and filamentous fungi. Therefore, this battery of enzymes most probably helps to facilitate the complex blueprint of lysis, branching, and cross-linking that involves the glucan layers of the fungal cell wall (Adams 2004).

According to Chet and Inbar (1994), cell wall of the most of fungal species hydrolases characterized have chitinase or glucanase, and these enzymes also exhibit the transglycosylase activity. Therefore, there is possible contribution to breakage, re-forming, and re-distribution of bonds between and within polymers leading to possible re-molding of the cell wall during fungal cell-wall development and morphogenesis.

Hence, the cell wall is an essential component to the cell and provides one of major defencing target to study. Currently, little is known about the cell wall of different species of filamentous fungi (Kils et al. 2002). For better understanding of the fungal cell wall and of its adaptation to various conditions, some more experimental validations are required to understand the defencing property adaptations to the external environment. The cell wall is a highly dynamic structure and able to adapt to various changes, either environmental (e.g., heat, pH, osmolarity, and chemical compounds), developmental (e.g., mating, growth, budding, branching, and sporulation), or genetic (e.g., mutations in cell-wall-related genes) (Kollar et al. 1997; Bowman and Free 2006).

In this experimental study, S-CCI protocol is proved to be the best compared to the existing DNA extraction protocol, because, technically, S-CCI is simple, work faster, less prone to cross contamination, and inexpensive than other methods. Samples were also amenable to PCR amplification and can be used routinely to identify and screening of fungal pathogens in both clinical and laboratory settings.
